# The mediating role of emotional intelligence between self-efficacy and resilience in Chinese secondary vocational students

**DOI:** 10.3389/fpsyt.2024.1382881

**Published:** 2024-07-09

**Authors:** Ruichen Jiang

**Affiliations:** ^1^ School of Teacher Education, Anqing Normal University, Anqing, China; ^2^ Institute of Educational Neuroscience, Anqing Normal University, Anqing, China; ^3^ Affective Computing and Intelligent Learning Cognitive Psychology Experimental Center, Anqing Normal University, Anqing, China

**Keywords:** secondary vocational students, self-efficacy, emotional intelligence, resilience, survey

## Abstract

**Purpose:**

This study aimed to explore the relationship between self-efficacy and resilience in Chinese secondary vocational students and examine the mediating effect of emotional intelligence.

**Methods:**

In September 2023, a cross-sectional survey was conducted in 282 Chinese students from three secondary vocational schools by using a voluntary and anonymous structured questionnaire, which included a general self-efficacy scale (GSES), emotional intelligence scale (EIS), and resilience scale (RS). The data were analyzed using SPSS 26.0 software and macro PROCESS.

**Results:**

The scores of self-efficacy, emotional intelligence, and resilience of Chinese secondary vocational students were above the average level. Correlations among the self-efficacy, emotional intelligence, and resilience levels of students were significant. The analyses of mediating effect showed that emotional intelligence partially mediated the influence of self-efficacy on resilience of secondary vocational students.

**Conclusion:**

Self-efficacy was positively associated with resilience. Self-efficacy not only has a direct effect on the resilience of secondary vocational students but it also indirectly affects the resilience through the mediating role of emotional intelligence. These findings valuable for designing the secondary vocational school programs aimed at improving students’ psychological resilience.

## Introduction

With the rapid development of Chinese economy, the demand for skilled professionals has become increasingly prominent (1). In this context, the number of secondary vocational schools has been steadily increasing, making secondary vocational students a significant part of China's youth. Professional training provided during vocational education equips young secondary vocational school students with necessary skills and competencies that are immediately valuable in the workplace and can reduce the risk of unemployment in the early years when teenagers enter the labor market (2). In 2022, the number of secondary vocational schools (including technical schools) in China was 7,201, enrolling 4,847,800 students, which accounts for 33.85% of the total number of students enrolled in senior high schools. In addition, the mental health condition of this group is of great concern to the public because of the challenges and dilemma they encounter in their study and life (3). Compared with ordinary high school students, secondary vocational students always experience family and social prejudice and are more prone to mental health problems (4, 5). Lu used the Chinese Secondary School Students Mental Health Scale (MSSMHS) to investigate 833 students from secondary vocational schools in X County, Guizhou Province, and found that the mental health problems of secondary vocational students include mainly obsessive tendency, anxiety, emotional instability, learning pressure, interpersonal relationship, and other factors (6). Thus, there is an urgent need to focus on the mental health status of secondary vocational students.

Resilience is an important mental resource for an individual to maintain positive adaptation in the face of challenging events and adverse situations. When facing problems in parent–child relationship or interpersonal relationships, resilient individuals tend to adopt more confrontational, proactive, and problem-solving coping strategies. In addition, resilience is also positive psychological resource to make the vocational students more employable (2). Thus, providing training and developing resilience among secondary vocational students have become crucial for improving their mental health and career development.

According to Bandura’s social cognitive theory, self-efficacy is a subjective self-evaluation and belief derived from an individual’s own experiences (7), that is, the result of an individual’s evaluation of whether they can successfully complete a certain achievement behavior, which could be divided into three dimensions: magnitude, strength, and generality (8); After the self-assessment, an individual tends to set appropriate goals based on the evaluation results and determine the plan to achieve them (9). Previous studies have evidenced that adolescents with high self-efficacy are self-confident and less susceptible to mental disorders when they encounter setbacks, and show higher academic performance (10, 11). Andriani et al. showed that self-efficacy has a positive relationship with learning motivation (8). Strengthening self-efficacy can also improve the learning outcomes of vocational education students (12).

Emotional intelligence refers to a series of non-cognitive abilities and skills for an individual to recognize and monitor their or others’ emotions and was proved to be positively correlated with self-efficacy (13, 14), mental health (15, 16), job performance (17), and positive adolescent development (12, 13). Numerous studies have suggested that emotional intelligence has a good predictive effect on resilience, stating that resilient people better understand and manage their emotions, which could also be related to higher levels of emotional intelligence (13, 18, 19). Teenagers are in a critical period of personality development, and their emotional state directly affects their mental health and future development (20). Secondary vocational students with a higher level of emotional intelligence can be more resilient (21).

According to the society-to-cells resilience theory (22), emotional regulation ability and optimism are important characteristics for building mental resilience. Therefore, it seems reasonable to infer that there may be potential interactions between self-efficacy, emotional intelligence and resilience.

Taken together, the relationship between self-efficacy and resilience of secondary vocational students has attracted the attention of researchers. Emotional intelligence, self-efficacy, and resilience appear to be highly correlated with each other in theory (23–25), however, the mechanism of influence of self-efficacy on emotional intelligence and resilience remains unclear. On the one hand, self-efficacy seems to be a predictor of emotional intelligence, and emotional intelligence is the individual’s ability to control emotions and could provide a cognitive judgment to regulate self-efficacy. On the other hand, according to the related theoretical framework of resilience, self-efficacy is one of the important protective factors of resilience (26). In addition, a significant positive correlation between emotional intelligence and resilience has been demonstrated (14, 27). Against this backdrop, this study attempts to identify the effect of self-efficacy on the resilience of secondary vocational students and the possible mediating role of emotional intelligence between these two variables, aiming to provide a reference for preparing the school program to improve secondary vocational students’ psychological resilience. Based on this, we hypothesized that. 1) self-efficacy, emotional intelligence, and resilience of secondary vocational school students would be correlated. 2) the self-efficacy of secondary vocational school students would predict resilience. 3) emotional intelligence would play a mediating role between self-efficacy and resilience in secondary vocational school student.

## Materials and methods

Special Committee for Scientific Research and Academic Ethics of Anqing Normal University reviewed and approved this study. Informed consent was obtained from the study participants or their guardians before the study began, and guidelines outlined in the Declaration of Helsinki were followed.

### Sampling and participants

The sample size calculator can be freely accessed at www.raosoft.com/samplesize.html. A total of 282 subjects from three secondary vocational schools in Anhui Province of China were selected as the objects of investigation by using the cluster sampling method. After obtaining informed consent from the subjects, or their guadians the participants were tested in a group using unified guidance language, and the questionnaires were uniformly collected. A total of 281 (99.65%) valid questionnaires were collected. After eliminating unusable ones (such as answers with a missing rate of over 50%, incomplete information or regular answers), 267 valid questionnaires were retained, with a valid rate of 94.68%. The students’ average age was 16.35 years (SD = 1.58), ranging from 14 to 20 years. Among them, 128 were male students, accounting for 47.94%; 157 students were from the rural area, accounting for 58.80%; and 124 students had good family relationship, accounting for 46.44% of the total.

### Socio-demographic information

The socio-demographic information collected in this study included age, gender, grade, birthplace (urban vs. rural), and family relation (good vs. bad).

### Research instruments

The structured questionnaire consisted of three separate scales developed or modified by local researchers in China and were suitable for assessing the subjects of this study.

### General self-efficacy scale

The general self-efficacy scale (GSES) was developed by Ralf Schwarzer, and it was later revised and adopted to Chinese culture by Wang (28). It was used to check the overall self-evaluation of secondary vocational students (29). The scale contains 10 items, such as “I can always solve problems if I try my best. ” It uses Likert’s four-point scoring method, with “1” denoting “not at all true,” “2” denoting “somewhat incorrect,” “3” denoting “mostly true,” and “4” denoting “exactly true.” A higher score implied higher self-efficacy. The value of Cronbach’s α of this scale was.88. The prediction validity of GSES was tested by using anxiety as criterion. There was a significant negative correlation between GSES and trait anxiety, state anxiety and test anxiety (TAS), and the correlation coefficients were -.301,.422 and.253, respectively.

### Emotional intelligence scale

The emotional intelligence scale (EIS) was developed by Schutte et al. in 1988 and later translated and revised by Liu (30). This scale has been widely used in China (31, 32) and consists of 21 items, such as “I can always solve problems if I try my best,” and four dimensions, namely regulation of perceived emotions, self-emotion management, others’ emotion management, and emotion application. Likert’s five-point scoring method was adopted, with “1” representing “strongly disagree” and “5” representing “strongly agree.” A higher score implied a higher level of emotional intelligence. The value of Cronbach’s α coefficient of this scale was.92. The pairwise correlation among the factors was significant, and the correlation coefficient was between .294 and .462, which was a low to moderate positive correlation. The correlation coefficient between the factors and the total score was ranging from .616 to .800, which denote strong positive correlation, indicating that each factor was consistent with the overall concept.

### Resilience scale of adolescents

The Resilience Scale of Adolescents (RS) developed by Hu and Gan (33) was used in this study (33). This scale has been widely used in China (34) and comprises a total of 27 items, such as “My life has a clear purpose, “ and 5 dimensions, including goal concentration, emotional control, positive cognition, family support, and interpersonal assistance (34). It uses Likert’s five-point scoring method, with 1 indicating completely inconsistent, 2 indicating inconsistent, 3 indicating not sure, 4 indicating consistent, and 5 indicating completely consistent. A higher score implied a higher level of resilience. The internal consistency coefficient of the scale was .88, and the internal consistency coefficient of the subscale was ranging from.69 -.83. The pairwise correlation among the factors was significant, and the correlation coefficient was between.12 and.56, which was a low to moderate positive correlation. The correlation coefficient between the factors and the total score was ranging from.54 to.73, which was medium to high positive correlation, indicating that each factor was consistent with the overall concept.

### Statistical analysis

SPSS26.0 and macro PROCESS were used for data processing and analysis. Descriptive statistical analysis, independent sample *t* test, Pearson correlation analysis, regression analysis, and mediation effect tests were the statistical methods used in this study.

Before the analysis, all data were tested for normality and were found to fulfill the criteria. The skewness coefficient method was used to test whether the scores obtained show normal distribution (35). The skewness values obtained for different scales used in this study were -.445 for the “General Self-efficacy Scale”, “-.728” for the “Emotional Intelligence Scale,” and “.365” for the “Resilience Scale of Adolescents.” The normally distributed data are expressed as the mean ± standard deviation, and the numerical data are expressed as *n*.

## Results

### Common method variance

Following the recommendations of Williams and McGonagle (36), Harman’s single-factor test was used for determining the common method variance bias. Variance was found to be less than the threshold of <25%, indicating that common method variance was not present (36).

### Comparison of total scores of self-efficacy, emotional intelligence, and resilience in secondary vocational students with different demographic variables

The scores of self-efficacy, emotional intelligence, and resilience of Chinese secondary vocational students were above the average level. The total score of resilience and emotional intelligence did not differ between the male and female students. The self-efficacy score of boys was significantly higher than that of girls (*t* = 2.77, *p < 0.01*). The scores of self-efficacy (*t* = -2.80, *p < 0.01*) and resilience (*t* = -3.48, *p < 0.01*) of urban students were significantly higher than those of students from rural areas. In addition, the total scores of resilience (*t* = 3.12, *p* < 0.01) and self-efficacy (*t* = 4.38, *p* < 0.001) of secondary vocational students with different family relationships differed significantly (**Table 1**).

**Table 1:** Result of inferential statistics.

Variable	Mean	Gender			Birthplace			Family relation		
		Male (128)	Female (139)	t	Rural (157)	Urban (110)	t	Good (124)	Bad (143)	t
Self-efficacy	27.68	28.34±3.31	27.07±4.21	2.77**	27.13±3.95	28.46±3.57	-2.80**	28.75±3.47	26.75±3.92	4.38***
Emotional intelligence	78.42	79.34±8.34	77.58±8.58	1.70	77.83±8.44	79.26±8.54	-1.35	79.24±8.54	77.71±8.43	1.48
Resilience	89.44	90.27±8.45	88.67±9.25	1.48	87.89±9.18	91.65±8.02	-3.48**	91.23±8.88	87.88±8.65	3.12**

**p < 0.01; ***p < 0.001.

### Correlation analysis of the studied variables

As shown in **Table 2**, Pearson’s product difference analysis was conducted between self-efficacy, emotional intelligence, and resilience of secondary vocational students. The result confirmed hypothesis 1 and revealed an obvious correlation among the three variables (*p* < 0.01).

**Table 2:** Correlation analysis of self-efficacy, emotional intelligence, and resilience of secondary vocational students.

Variable	1	2	3	4	5	6	7	8
1 Self-efficacy	–	0.533**	0.190**	0.250**	0.185**	0.246**	0.236**	0.403**
2 Emotional intelligence	0.533**	–	0.299**	0.213**	0.234**	0.183**	0.200**	0.407**
3 goal concentration	0.190**	0.299**	–	-0.134*	0.413**	0.075	-0.171**	0.392**
4 emotional control	0.250**	0.213**	-0.134*	–	-0.125*	0.315**	0.483**	0.641**
5 positive cognition	0.185**	0.234**	0.413**	-0.125*	–	-0.013*	-0.015	0.368**
6 family support	0.246**	0.183**	0.075	0.315**	-0.013	–	0.421**	0.655**
7 interpersonal assistance	0.236**	0.200**	-0.171**	0.483**	-0.015	0.421**	–	0.664**
8 Resilience	0.403**	0.407**	0.392**	0.641**	0.368**	0.655**	0.664**	

*p < 0.05, **p < 0.01.

### Mediating role of emotional intelligence between self-efficacy and resilience

Regression analysis was used to test whether emotional intelligence has a mediating effect. Three models were used to analyze the mediating effect: Model 1, regression analysis of the independent variable (X) and the dependent variable (Y); Model 2, regression analysis of the independent variable (X) and the mediating variable (M); Model 3, regression analysis of the independent variable (X), mediating variable (M), and dependent variable (Y). It was assumed that if X affects Y by affecting M, then M is the mediating variable. Self-efficacy was considered as the independent variable (X), level of resilience was the dependent variable (Y), and emotional intelligence ability was assumed to be the intermediary variable (M). The regression analysis was performed, and the results are shown in **Table 3**.

**Table 3:** Mediating role of emotional intelligence between self-efficacy and resilience.

Criterion variable	Predictor variable	Model fit indices			Parameter significance estimation	
		R	R^2^	F	β	*t*
Resilience	self-efficacy	0.403	0.162	51.383***	0.932	7.168***
Emotional intelligence	self-efficacy	0.407	0.166	52.562***	0.426	7.250***
Resilience	self-efficacy	0.463	0.214	35.935***	0.602	4.035***
	Emotional intelligence				0.281	4.162***

***p < 0.001.

**Figure 1:**
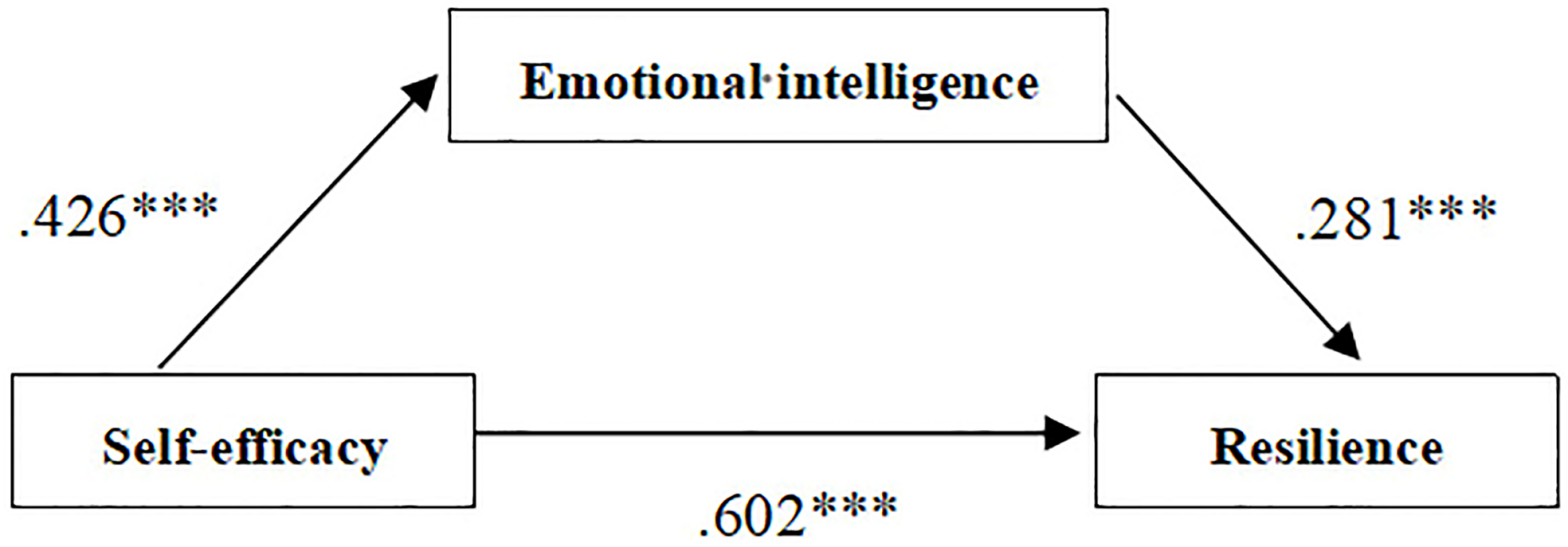
The mediating role of emotional intelligence. The number represents the regression coefficient of the two variables connected by the arrow line. ***p < 0.001.

Self-efficacy had a significant predictive effect on resilience and confirmed hypothesis 2 (*β* = .932, *t* = 7.168, *p* < 0.001), and this result was true even after addition of the intermediary variable emotional intelligence (*β* = .602, *t* = 4.035, *p* < 0.001). Self-efficacy was a significant predictor of emotional intelligence (*β* = .426, *t* = 7.250, *p* < 0.001), and emotional intelligence was a significant predictor of resilience (*β* =. 281, *t* = 4.162, *p* < 0.001). The confidence intervals were estimated then by Bootstrap method with deviation correction. The results showed that 95% confidence intervals did not contain 0, indicating that the mediating effect was significant, which validated Hypothesis 3. Self-efficacy of secondary vocational students can directly predict resilience. Resilience can also be affected through the mediating effect of emotional intelligence, and the proportion of intermediary effect was 16.59 %. According to the test results, the intermediary role model was constructed in this study, as shown in **Figure 1**.

## Discussion

### Status of self-efficacy, emotional intelligence, and resilience of secondary vocational students

The self-efficacy of secondary vocational students was above the medium level, which is consistent with the findings of previous studies (12, 29). Compared with junior high school students, learning pressure among secondary vocational students is significantly low. Practical ability and employment-oriented learning content can mobilize students' learning enthusiasm, and therefore, secondary vocational students' learning interest was found to be significantly improved. All these factors contribute to the improvement of secondary vocational students' sense of self-efficacy.

The emotional intelligence of secondary vocational students was above the medium level. This result is in close agreement with that of a previous study conducted by Liu (29). Studies have shown that a variety of internal protective factors (such as emotional control ability and self-efficacy) and external environmental factors (such as relatives and peers) may affect the level of resilience of individuals (37). Moreover, the resilience of secondary vocational students was above the medium level, which is consistent with previous research (29, 38). Secondary vocational students are shunted to vocational schools after graduation from junior high school, and they may face more challenges in life (39). Having faced examinations such as the senior high school entrance examination and various setbacks in life, along with the accumulated personal experience and improved environmental adaptability, secondary vocational students tend to exhibit significantly higher levels of resilience

We also found that the level of resilience of secondary vocational students with good family relationships was significantly higher than that of secondary vocational students with bad family relationships, which is consistent with some previous research results (6). Family is the first and most important place for individuals’ growth, and therefore, family environment and atmosphere play a crucial role in cultivating an individual’s resilience. A good family relationship can enable secondary vocational students to obtain safety and love.

### Relationship between self-efficacy, emotional intelligence, and resilience of secondary vocational students

This study provides evidence that higher levels of general self-efficacy predict higher levels of resilience, which is consistent with previous research findings (40, 41). Specifically, based on the results of previous studies and this study on secondary vocational school students, we determined that self-efficacy has a positive impact on the resilience of secondary vocational school students. Students with high levels of self-efficacy tend to be confident about their abilities and resources for overcoming challenges (23) and tend to develop different resilient behavioral responses to cope with stress and other negative experiences (40).

Research suggests that self-efficacy was positively correlated with emotional intelligence of secondary vocational school students, indicating its role as an important internal driving factor for promoting emotional intelligence of secondary vocational school students. This is in accordance with the results of previous studies (29, 40), which verified that a high level of self-efficacy could enhance students’ ability to manage emotions and deal with stress. Higher levels of self-efficacy implied higher levels of emotional intelligence of secondary vocational school students. Strengthening self-efficacy and emotional intelligence can improve the learning outcomes of vocational education students (40).

We also determined the impact of emotional intelligence on the resilience of secondary vocational students. Secondary vocational students with high emotional intelligence tend to be confident and adopt a positive coping style when dealing with stressful life events, implying that they tend to be more resilient in life (40).

Interestingly, this study revealed that emotional intelligence serves as a partial mediating factor for the influence of self-efficacy on secondary vocational students’ resilience. These results confirm that self-efficacy influences secondary vocational school students’ resilience not only directly but also indirectly through emotional intelligence. Therefore, interventions that improve emotional intelligence as a strategy to enhance self-efficacy for promoting resilience are needed. The present study results also indicate that emotional intelligence plays an important role in enhancing secondary vocational students’ resilience, in agreement with the results of previous studies. Liu (2019) conducted a study on emotional intelligence and resilience of secondary vocational students and determined that there was a significant correlation between the two variables (29). Armstrong et al. studied the relationship between emotional intelligence and resilience and observed a strong correlation between them (21). Therefore, promoting the level of psychological resilience of secondary vocational students by improving their self-efficacy can be a promising strategy. Improving self-efficacy can also promote individuals’ emotional perception and positive regulation ability, thereby contributing to a positive change and enhancing resilience among secondary vocational students.

### Implications for education

The results of this study highlight the necessity to design and implement intervention programs and active measures focused on self-efficacy and emotional intelligence, aiming to help secondary vocational school students to develop high resilience levels. In addition to daily learning activities, secondary vocational students should be encouraged to participate actively in outdoor activities, such as physical exercise, which can help them improve their self-confidence and self-efficacy, and further strengthen their psychological well-being (42). Administrators and teachers in secondary vocational schools should pay attention to cultivating the students' abilities, guide them to focus on their strengths, and strive to create a platform to display their talents. Finally, warm and friendly family atmosphere is a protective factor for the psychological development of adolescents. Family members should pay attention to the shaping of a good family environment in their daily life, which is conducive to the healthy development of secondary vocational school students.

### Limitations and suggestions for future research

Several limitations of this study should be noted. First, the number of participants in the study is small. Secondly, the cross-sectional method was used for data collection from participants at a single point in time. In future studies, we can consider using longitudinal research methods to explore the continuous relationship between these different variables. In addition, the formation of resilience is not only affected by individual factors, but also by external factors such as family and school. In the follow-up research, other external variables can be included to achieve a deeper understanding of the development of psychological resilience of secondary vocational students.

## Conclusion

We clarified the relationship between self-efficacy, emotional intelligence, and resilience of secondary vocational school students, and the key findings can be summarized are as follows:

(1) There is a pairwise positive correlation among self-efficacy, emotional intelligence, and resilience;
(2) The self-efficacy of secondary vocational school students could predict resilience;
(3) Emotional intelligence plays a partial mediating role in the influence of secondary vocational students’ self-efficacy on their resilience.

## Data availability statement

The original contributions presented in the study are included in the article/**Supplementary Material**. Further inquiries can be directed to the corresponding author.

## Ethics statement

The studies involving humans were approved by Special committee for scientific research and academic ethics of Anqing Normal University. The studies were conducted in accordance with the local legislation and institutional requirements. The participants or their guardians provided their written informed consent to participate in this study.

## Author contributions

RJ: Conceptualization, Data curation, Formal analysis, Funding acquisition, Investigation, Methodology, Project administration, Resources, Software, Supervision, Validation, Visualization, Writing – original draft, Writing – review & editing.
